# Different Clinicopathological Characteristics and Prognostic Factors for Occult and Non-occult Breast Cancer: Analysis of the SEER Database

**DOI:** 10.3389/fonc.2020.01420

**Published:** 2020-08-19

**Authors:** Kai-Yan Huang, Jie Zhang, Wen-Fen Fu, Yu-Xiang Lin, Chuan-Gui Song

**Affiliations:** ^1^Department of Breast Surgery, Fujian Medical University Union Hospital, Fuzhou, China; ^2^Department of General Surgery, Fujian Medical University Union Hospital, Fuzhou, China

**Keywords:** occult breast cancer, breast cancer-specific survival, overall survival, SEER database, prognostic factor

## Abstract

**Purpose:** The aim of our study was to evaluate the different clinicopathological characteristics and prognostic factors for occult and non-occult breast cancer.

**Methods:** 572 OBC cases and 117,217 non-OBC patients between 2004 and 2015 was selected from Surveillance, Epidemiology, and End Results (SEER) database. We analyzed the clinicopathological characteristics and survival outcomes between OBC and non-OBC patients. Furthermore, the propensity score matching method was utilized to reduce the influences of baseline differences in demographic and clinical characteristics on outcome differences. Univariable and multivariable analyses were used to evaluate the prognostic factors of OBC patients.

**Results:** Compared with non-OBC patients, OBC patients in this study presented a higher proportion of older age, American Joint Committee on Cancer (AJCC) N3 stage, estrogen receptor (ER)-negative status, progesterone receptor (PR)-negative status, and human epidermal growth factor receptor-2 (HER-2)-positive status, and underwent more chemotherapy. Multivariate analysis revealed a better survival in overall patients with OBC patients according to breast cancer-specific survival (BCSS) and overall survival (OS). Propensity score analysis also achieved a similar result for OBC patients. Stratified analyses by nodal status and molecular subtypes indicated that these survival advantage were mainly presented in patients with AJCC N2/N3 stage or hormone receptor (HR)-positive tumors. In addition, nodal status, HER-2 status, and radiation status were demonstrated to be three independent prognostic factors for OBC patients.

**Conclusion:** Patients with OBC retained exclusive clinical characteristics and were shown to have a better outcome compared with non-OBC patients, especially for those with N2/N3 stage or HR-positive tumors.

## Introduction

Occult breast cancer (OBC) is a rare type of breast cancer which generally presents as axillary lymph node metastases without identified primary breast lesion. It is reported to be only account for 0.3–1.0% of all breast cancers, with a peak incidence at about 55 years old (1–5). Although it is generally believed that OBC has a similar biological behavior compared with node-positive non-OBC, the clinicopathological characteristics of this disease are still unclear (6–8). Some previous studies have indicated that the estrogen receptor (ER) status, triple negative subtype, and at least four positive lymph nodes were individual prognostic factors for occult breast cancer (6, 9–11). The survival outcomes of patients with OBC are also controversial. Compared with non-OBC cases, OBC patients were shown to have a similar or less unfavorable outcome (12, 13), while the others have drawn contrary conclusions (14, 15).

Up to now, the majority of the studies on OBC only consisted of a small, single-institution sample size. Prospective randomized trials have also not been performed due to the rarity of these patients. Ge et al. (13) conducted a study based on large population database (the Surveillance, Epidemiology, and End Results, SEER) to evaluate the survival difference between occult and non-occult breast cancer patients. The other SEER-based study by Johnson et al. (16) assessed the effect of local therapy and other clinical variables on risk of breast cancer-specific mortality in OBC patients. However, these two studies mainly focused on the importance of local regional treatment (type of surgery or radiation) in OBC. The clinicopathological characteristics and prognostic factors between OBC and non-OBC patients in different tumor stages and molecular subtypes have not been fully elucidated. Therefore, we conducted this study based on SEER database with newest data from 2004 to 2015 and utilize with propensity matching score method, aiming to better demonstrate the different clinicopathological characteristics and prognostic factors (especially the nodal status and molecular subtypes) between OBC and non-OBC patients.

## Materials and Methods

### Compliance With Ethical Standards

In the United States, cancer is a reportable disease. So we did not need to get patient consent. The SEER database is available to public. We signed a Data-Use Agreement for the SEER 1973–2015 Research Data File to get access conditions.

### Data Source and Study Population

We used SEER^*^Stat version 8.3.5 to generate a case list. We enrolled 117,789 patients according to the following inclusion criteria: female; year of diagnosis from 2004 to 2015; age of diagnosis between 20 and 79 years; breast cancer as the only primary malignant cancer diagnosis; unilateral cancer; American Joint Committee on Cancer (AJCC) sixth edition stage T0–T3; AJCC N1–N3; one or more positive LNs. Patients who present with distant metastasis, *in situ* disease, or had not undergone surgery were expelled from the study. Patients with stage T0N1–3M0 were defined as OBC and stage T1–3N1–3M0 were defined as non-OBC. We calculated follow-up durations from January 1, 2004 to December 31, 2015. Of 117,789 patients enrolled, 572 patients were diagnosed as OBC and 117,217 patients as non-OBC. Patient characteristics and treatment courses in our study were identified, including age, race, year of diagnosis, marital status, mastectomy or breast conserving surgery (BCS), radiotherapy and chemotherapy status. Tumor characteristics included laterality, tumor grade, AJCC stage, estrogen receptor (ER) status, progesterone receptor (PR) status, human epidermal growth factor receptor-2 (HER-2) status, and tumor subtype. For the HER-2 status was not recorded in the SEER database until 2010, the variable was not available or could not be used by some patients. The definition of breast subtype was as follows: Her2+/HR+ (ER and/or PR positive, HER2 positive), Her2+/HR– (ER and PR negative, HER2 positive), Her2-/HR+ (ER and/or PR positive, HER2 negative), Triple negative (ER, PR, and HER2 negative).

### Outcome Measurement

In our study, breast cancer-specific survival (BCSS) was used as a primary study outcome. It was calculated from the date of diagnosis to the date of death that due to breast cancer. Overall survival (OS), served as secondary outcome, was defined as from the date of diagnosis to the date of death, whether the patient died of breast cancer or not. Patients who were alive on the date of last contact were censored.

### Statistical Analysis

The chi-square test were conducted to describe the demographic and clinical characteristics of the OBC and non-OBC cases, in both the whole groups and 1:1 propensity score matched groups. The Kaplan–Meier method was utilized to generate the survival curves while the log-rank test was conducted to identify whether the differences in BCSS or OS rates between OBC patients and non-OBC patients was statistically significant. Hazard ratio (HR) with 95% confidence intervals (CI) was calculated by using a Cox proportional hazard regression model to determine the outcome-related factors. Factors with a *P*-value of 0.05 or less in univariate analysis were included as candidate variables in the multivariate analysis. These statistical analyses were conducted by using SPSS version 24.0 and R software version 3.6.1. In order to reduce the influences of baseline differences in demographic and clinical characteristics on outcome differences, the psmatch2 module were used to perform propensity score matching (17) in Stata version 13.0. The command matched OBC patients to non-OBC patients were with the following factors: age, year of diagnosis, race, marital status, grade, laterality, AJCC N status, ER status, PR status, HER-2 status, mastectomy or not, chemotherapy status, radiation status. All statistical analyses were two-sided, and a *P*-value of < 0.05 was considered as a significance level.

## Results

### Demographics and Clinical Characteristics of the Study Population

Overall, 117,789 eligible patients were enrolled in our study, including 572 cases of OBC and 117,217 cases of non-OBC. The median follow-up time was 63 months. The baseline characteristics of the OBC and non-OBC are summarized in Table 1. There were significant differences in characteristics between OBC and non-OBC cases, including age, grade, nodal status, ER status, PR status, HER-2 status, breast subtype, type of surgery, and chemotherapy status. The OBC patients presented a higher proportion of older age (50–79 years old, 76.2 vs. 65.2%, *p* < 0.001), unknown grade (71.9 vs. 3.3%, *p* < 0.001), N3 stage (17.3 vs. 8.5%, *p* < 0.001), ER-negative status (38.6 vs. 20.3%, *p* < 0.001), PR-negative status (54.2 vs. 30.5%, *p* < 0.001), HER-2-positive status (15.4 vs. 9.8%, *p* < 0.001), and chemotherapy status (83.9 vs. 75.8%, *p* < 0.001). In addition, the OBC patients appeared to be not inclined to accept mastectomy than non-OBC patients (35.3 vs. 56.6%, *p* < 0.001). Other characteristics, including year of diagnosis, race, marital status, laterality, AJCC stage, and radiation status, were similarly distributed between OBC and non-OBC patients.

**Table 1:** Baseline characteristics of patients with OBC and non-OBC.

**Characteristics**		**OBC (*****n*** **=** **572)**		**Non-OBC (*****n*** **=** **117,217)**		**Total (*****n*** **=** **117,789)**		***P*^**c**^**
		**No**	**%**	**No**	**%**	**No**	**%**	
**Median follow-up (months) (IQR)**		**61.5 (30–100.75)**		**63 (33–102)**		**63 (33–102)**		
Year of diagnosis	2004–2009	254	44.4	55,784	47.6	56,038	47.6	0.128
	2010–2015	318	55.6	61,433	52.4	61,751	52.4	
Age (years)	20–49	136	23.8	40,798	34.8	40,934	34.8	**<0.001**
	50–79	436	76.2	76,419	65.2	76,855	65.2	
Race	White	450	78.7	91,273	77.9	91,723	77.9	0.536
	Black	76	13.3	14,957	12.8	15,033	12.8	
	Other^a^	46	8.0	10,987	9.4	11,033	9.4	
Marital status	Married	340	59.4	72,521	61.9	72,861	61.9	0.233
	Not married^b^	232	40.6	44,696	38.1	44,928	38.1	
Laterality	Left	306	53.5	59,336	50.6	59,642	50.6	0.170
	Right	266	46.5	57,881	49.4	58,147	49.4	
Grade	I and II	29	5.1	62,896	53.7	62,925	53.4	**<0.001**
	III and IV	132	23.1	50,422	43.0	50,554	42.9	
	Unknown	411	71.9	3,899	3.3	4,310	3.7	
AJCC stage	II	368	64.3	78,516	67.0	78,884	67.0	0.179
	III	204	35.7	38,701	33.0	38,905	33.0	
Nodal status	1 to 3	368	64.3	86,604	73.9	86,972	73.8	**<0.001**
	4 to 9	105	18.4	20,636	17.6	20,741	17.6	
	>9	99	17.3	9,977	8.5	10,076	8.6	
ER status	Positive	302	52.8	90,613	77.3	90,915	77.2	**<0.001**
	Negative	221	38.6	23,750	20.3	23,971	20.4	
	Others	49	8.6	2,854	2.4	2,903	2.5	
PR status	Positive	202	35.3	77,786	66.4	77,988	66.2	**<0.001**
	Negative	310	54.2	35,779	30.5	36,089	30.6	
	Others	60	10.5	3,652	3.1	3,712	3.2	
HER-2 status	Positive	88	15.4	11,478	9.8	11,566	9.8	**<0.001**
	Negative	185	32.3	47,530	40.5	47,715	40.5	
	Others	299	52.3	58,209	49.7	58,508	49.7	
Breast subtype	Her2+/HR+	54	9.4	7,981	6.8	8,035	6.8	**<0.001**
	Her2+/HR-	32	5.6	3,477	3.0	3,509	3.0	
	Her2-/HR+	114	19.9	40,574	34.6	40,688	34.5	
	Triple negative	71	12.4	6,904	5.9	6,975	5.9	
	Unknown	301	52.6	58,281	49.7	58,582	49.7	
Mastectomy	Yes	202	35.3	66,315	56.6	66,517	56.5	**<0.001**
	No	370	64.7	50,902	43.4	51,272	43.5	
Chemotherapy	Yes	480	83.9	88,848	75.8	89,328	75.8	**<0.001**
	No/Unknown	92	16.1	28,369	24.2	28,461	24.2	
Radiation	Yes	320	55.9	65,740	56.1	66,060	56.1	0.769
	No	252	44.1	51,477	43.9	51,729	43.9	

OBC, occult breast cancer; non-OBC, non-occult breast cancer; AJCC, American Joint Committee on Cancer; ER, estrogen receptor; PR, progesterone receptor; HER-2, human epidermal growth factor receptor-2; HR, hormone receptor; IQR, interquartile range.

a Other includes American Indian/Alaskan native and Asian/Pacific Islander and Unknown.

b Not married includes divorced, separated, single (never married), unmarried or domestic partner, and widowed.

c *The P-value of the Chi-square test was calculated between the OBC and non-OBC groups, and bold type indicates significance*.

### Comparison of Survival Between OBC and Non-OBC Patients

As shown in Table 2, compared to overall patients with non-OBC, multivariate analysis revealed a better survival in overall patients with OBC according to BCSS and OS (HR = 0.646, 95% CI = 0.504–0.828, *p* = 0.001; HR = 0.709, 95% CI = 0.575–0.874, *p* = 0.001, respectively). We also stratified the different nodal stages to validate the different survival outcomes between OBC and non-OBC cases. In patients with N1 stage, the OBC patients demonstrated a better prognosis than non-OBC patients in terms of BCSS (HR = 0.661, 95% CI = 0.456–0.959, *p* = 0.029) but not OS. In patients with N2 stage, the OBC patients were indicated to have a better prognosis than non-OBC patients in terms of both BCSS and OS (HR = 0.542, 95% CI = 0.312–0.943, *p* = 0.030; HR = 0.607, 95% CI = 0.378–0.974, *p* = 0.039, respectively). While for patients with N3 stage, no statistical survival differences were observed between OBC and non-OBC patients. In the subgroup of N2/N3 patients, OBC had better survival compared with non-OBC (BCSS, HR = 0.651, 95% CI = 0.466–0.907, *p* = 0.011; OS, HR = 0.673, 95% CI = 0.499–0.908, *p* = 0.009).

**Table 2:** Comparison of breast cancer-specific survival (BCSS) and overall survival (OS) between OBC and non-OBC after subgroup analyses using a multivariate Cox proportional hazard model.

**Nodal status**	**BCSS**		**OS**	
	**HR (95% CI)**	***P*^**a**^**	**HR (95% CI)**	***P*^**a**^**
N1		**0.029**		0.077
OBC	0.661 (0.456–0.959)		0.767 (0.572–1.029)	
non-OBC	Reference		Reference	
N2		**0.030**		**0.039**
OBC	0.542 (0.312–0.943)		0.607 (0.378–0.974)	
non-OBC	Reference		Reference	
N3		0.147		0.090
OBC	0.735 (0.485–1.114)		0.716 (0.487–1.053)	
non-OBC	Reference		Reference	
N2/N3				
OBC	0.651 (0.466–0.907)	**0.011**	0.673 (0.499–0.908)	**0.009**
non-OBC	Reference		Reference	
N1/N2/N3		**0.001**		**0.001**
OBC	0.646 (0.504–0.828)		0.709 (0.575–0.874)	
non-OBC	Reference		Reference	

OBC, occult breast cancer; non-OBC, non-occult breast cancer; CI, confidence interval; HR, hazard ratio; BCSS, breast cancer-specific survival; OS, overall survival.

a *P-value was adjusted by a multivariate Cox proportional hazard regression model including age, race, marital status, year of diagnosis, grade, mastectomy, or not, ER status, PR status, HER-2 status, chemotherapy, and radiation. Bold type indicates significance*.

### Survival Estimates in Matched Groups

We conducted 1:1 (OBC/non-OBC) propensity score analysis between OBC and non-OBC patients by using a propensity score matching method and a comprehensive consideration of the confounding factors that affected breast cancer outcomes (Table 3). Finally, we obtained a group with 1,144 patients, and each subgroup included 572 patients. For the matched groups, no factors differed significantly between OBC and non-OBC patients.

**Table 3:** Baseline characteristics of patients with OBC and non-OBC in the propensity score matched group.

**Characteristics**		**OBC (*****n*** **=** **572)**		**Non-OBC (*****n*** **=** **572)**		**Total (*****n*** **=** **1,144)**		***P*^**c**^**
		**No**	**%**	**No**	**%**	**No**	**%**	
**Median follow-up (months) (IQR)**		**60.5 (30–100.75)**		**61.5 (32–101.75)**		**61 (31–101)**		
Year of diagnosis	2004–2009	254	44.4	260	45.5	514	44.9	0.721
	2010–2015	318	55.6	312	54.5	630	55.1	
Age (years)	20–49	136	23.8	142	24.8	278	24.3	0.679
	50–79	436	76.2	430	75.2	866	75.7	
Race	White	450	78.7	458	80.1	908	79.4	0.444
	Black	76	13.3	79	13.8	155	13.5	
	Other^a^	46	8.0	35	6.1	81	7.1	
Marital status	Married	340	59.4	346	60.5	686	60.0	0.717
	Not married^b^	232	40.6	226	39.5	458	40.0	
Laterality	Left	306	53.5	309	54.0	615	53.8	0.859
	Right	266	46.5	263	46.0	529	46.2	
Grade	I and II	29	5.1	29	5.1	58	5.1	1.000
	III and IV	132	23.1	132	23.1	264	23.1	
	unknown	411	71.9	411	71.9	822	71.9	
Nodal status	1 to 3	368	64.3	367	64.2	735	64.2	0.642
	4 to 9	105	18.4	96	16.8	201	17.6	
	>9	99	17.3	109	19.1	208	18.2	
ER status	Positive	302	52.8	304	53.1	606	53.0	0.656
	Negative	221	38.6	211	36.9	432	37.8	
	Others	49	8.6	57	10	106	9.3	
PR status	Positive	202	35.3	201	35.1	403	35.2	0.982
	Negative	310	54.2	309	54.0	619	54.1	
	Others	60	10.5	62	10.8	122	10.7	
HER-2 status	Positive	88	15.4	73	12.8	161	14.1	0.371
	Negative	185	32.3	200	35.0	385	33.7	
	Others	299	52.3	299	52.3	598	52.3	
Breast subtype	Her2+/HR+	54	9.4	43	7.5	97	8.5	0.729
	Her2+/HR–	32	5.6	29	5.1	61	5.3	
	Her2–/HR+	114	19.9	119	20.8	233	20.4	
	Triple negative	71	12.4	80	14.0	151	13.2	
	Unknown	301	52.6	301	52.6	602	52.6	
Mastectomy	Yes	202	35.3	210	36.7	412	36.0	0.622
	No	370	64.7	362	63.3	732	64.0	
Chemotherapy	Yes	480	83.9	490	85.7	970	84.8	0.410
	No/Unknown	92	16.3	82	14.3	174	15.2	
Radiation	Yes	320	55.9	317	55.4	637	55.7	0.945
	No	252	44.1	255	44.5	507	44.3	

OBC, occult breast cancer; non-OBC, non-occult breast cancer; AJCC, American Joint Committee on Cancer; ER, estrogen receptor; PR, progesterone receptor; HER-2, human epidermal growth factor receptor-2; HR, hormone receptor; IQR, interquartile range.

a Other includes American Indian/Alaskan native and Asian/Pacific Islander and Unknown.

b Not married includes divorced, separated, single (never married), unmarried or domestic partner, and widowed.

c *The P-value of the Chi-square test was calculated between the OBC and non-OBC groups*.

In addition, stratified analyses by nodal status and molecular subtype was also conducted to validate the different outcomes between OBC cases and non-OBC cases. Compared to overall patients with non-OBC, multivariate analysis revealed a better survival in overall patients with OBC according to BCSS and OS (HR = 0.697, 95% CI = 0.508–0.957, *p* = 0.025; HR = 0.753, 95% CI = 0.574–0.987, *p* = 0.040, respectively). For patients with N1 stage, no statistical survival differences were identified between OBC and non-OBC patients. While for patients with N2/N3 stage (shown in Table 4), the OBC patients demonstrated a better prognosis than non-OBC patients in terms of both BCSS and OS (HR = 0.604, 95% CI = 0.397–0.919, *p* = 0.019; HR = 0.620, 95% CI = 0.425–0.904, *p* = 0.013, respectively). As for different molecular subtype, the OBC cases with HR positive were indicated to have a better prognosis than non-OBC patients in terms of both BCSS and OS (Table 5, HR = 0.482, 95% CI = 0.290–0.801, *p* = 0.005; HR = 0.603, 95% CI = 0.397–0.916, *p* = 0.018, respectively). However, no significant statistical survival difference was observed for OBC and non-OBC patients in other subgroups.

**Table 4:** Comparison of breast cancer-specific survival (BCSS) and overall survival (OS) between OBC and non-OBC in different nodal status using a multivariate Cox proportional hazard model in the propensity score matched group.

**Nodal status**	**BCSS**		**OS**	
	**HR (95% CI)**	***P*^**a**^**	**HR (95% CI)**	***P*^**a**^**
N1		0.331		0.619
OBC	0.785 (0.481–1.280)		0.903 (0.605–1.349)	
non-OBC	Reference		Reference	
N2/N3		**0.019**		**0.013**
OBC	0.604 (0.397–0.919)		0.620 (0.425–0.904)	
non-OBC	Reference		Reference	
N1/N2/N3		**0.025**		**0.040**
OBC	0.697 (0.508–0.957)		0.753 (0.574–0.987)	
non-OBC	Reference		Reference	

OBC, occult breast cancer; non-OBC, non-occult breast cancer; HR, hazard ratio; CI, confidence interval; BCSS, breast cancer-specific survival; OS, overall survival.

a *P-value was adjusted by a multivariate Cox proportional hazard regression model including age, race, marital status, year of diagnosis, grade, mastectomy or not, ER status, PR status, HER-2 status, chemotherapy, and radiation. Bold type indicates significance*.

**Table 5:** Comparison of breast cancer-specific survival (BCSS) and overall survival (OS) between OBC and non-OBC in different hormone receptor status using a multivariate Cox proportional hazard model in the propensity score matched group.

**Cohorts**	**BCSS**		**OS**	
	**HR (95% CI)**	***P*^**a**^**	**HR (95% CI)**	***P*^**a**^**
HR (+)		**0.005**		**0.018**
OBC	0.482 (0.290,0.801)		0.603 (0.397,0.916)	
non-OBC	Reference		Reference	
HR (–)		0.252		0.276
OBC	0.766 (0.485,1.209)		0.800 (0.536,1.195)	
non-OBC	Reference		Reference	
HER-2 (+)		0.707		0.707
OBC	0.751 (0.169,3.345)		0.751 (0.169,3.345)	
non-OBC	Reference		Reference	
HER-2 (–)		0.802		0.954
OBC	0.931 (0.533,1.627)		0.986 (0.597,1.626)	
non-OBC	Reference		Reference	
TNBC		0.968		0.818
OBC	0.985 (0.461,2.105)		0.919 (0.446,1.891)	
non-OBC	Reference		Reference	
non-TNBC		0.640		0.985
OBC	0.842 (0.411,1.727)		0.994 (0.524,1.884)	
non-OBC	Reference		Reference	

OBC, occult breast cancer; non-OBC, non-occult breast cancer; HR, hormone receptor; HR, hazard ratio; HER-2, human epidermal growth factor receptor-2; TNBC, triple negative breast cancer; CI, confidence interval; BCSS, breast cancer-specific survival; OS, overall survival.

a *P-value was adjusted by a multivariate Cox proportional hazard regression model. Bold type indicates significance*.

**Figure 1:**
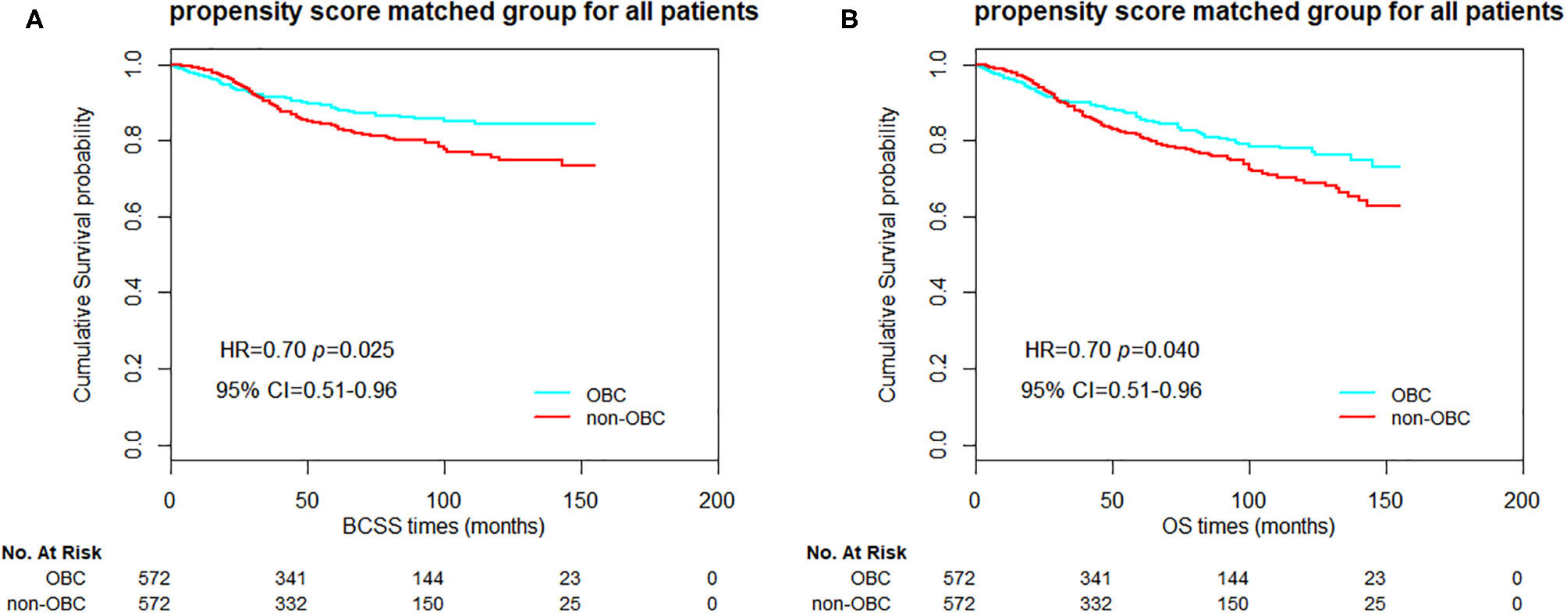
Kaplan–Meier curves of breast cancer-specific survival (BCSS) **(A)** and overall survival (OS) **(B)** based on two groups of propensity score matched patients.

**Figure 2:**
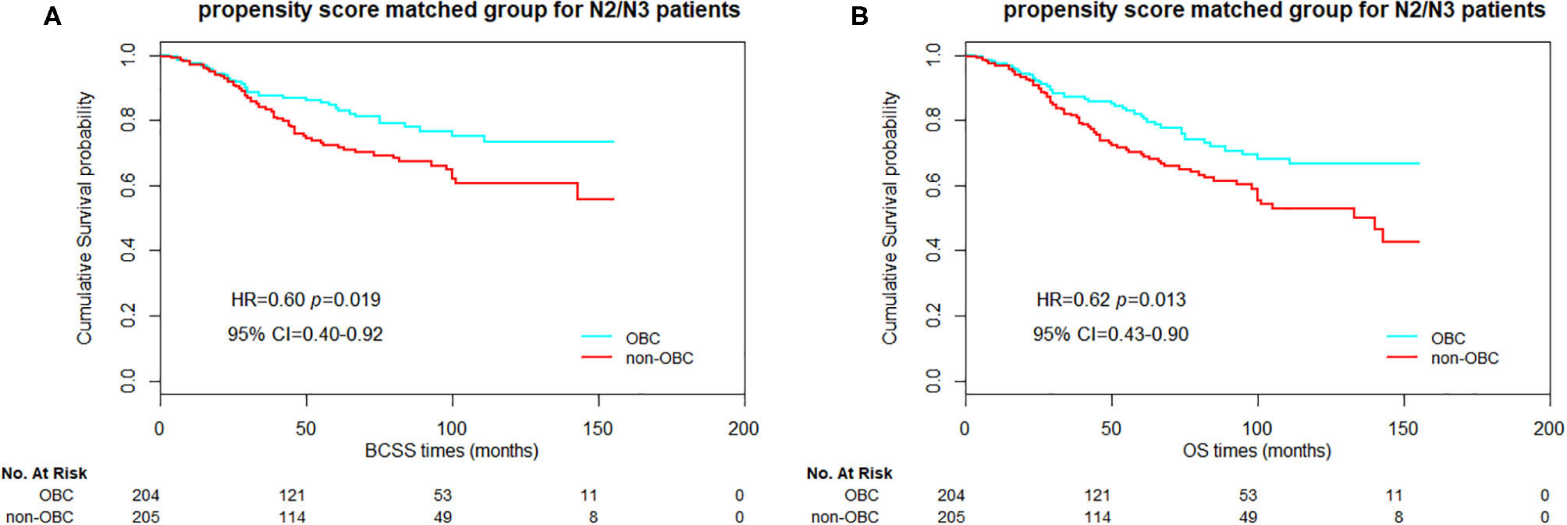
Kaplan–Meier curves of breast cancer-specific survival (BCSS) **(A)** and overall survival (OS) **(B)** based on two groups of propensity score matched N2/N3 patients.

**Figure 3:**
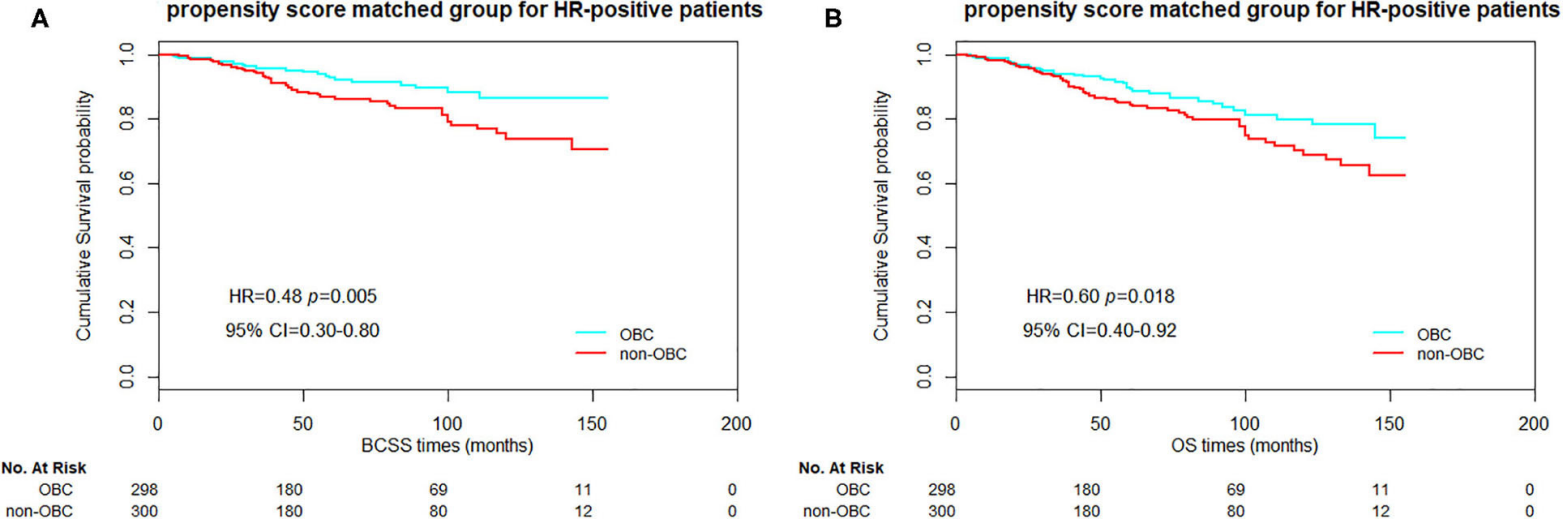
Kaplan–Meier curves of breast cancer-specific survival (BCSS) **(A)** and overall survival (OS) **(B)** based on two groups of propensity score matched HR-positive patients.

Furthermore, we compared the BCSS and OS between the 1:1 propensity score matched OBC cases and non-OBC cases as well. In overall matched patients, significant differences in BCSS and OS were observed between the groups (Figure 1, *p* = 0.025 and 0.040 for BCSS and OS, respectively). For N2/N3 patients, we also obtained a significant statistical difference in BCSS and OS (Figure 2, *p* = 0.019 and 0.013, respectively). As for HR-positive patients, we observed a significant statistical difference in BCSS and OS as well (Figure 3, *p* = 0.005 and 0.018, respectively).

### Analyses on the Outcome-Related Factors of OBC Using Cox Proportional Hazard Regression Models

We investigated the prognostic factors that related with BCSS and OS in the cohort of OBC patients. The univariate Cox regression analysis for each variable of OBC was shown in Supplementary Table S1. AJCC stage, nodal status, ER, PR, or HER-2 status and radiation status significantly correlated with BCSS and OS, thereby were selected into multivariate Cox regression analysis.

As shown in Table 6, nodal status, HER-2 status, and radiation status were three independent prognostic factors for OBC patients in the multivariate Cox regression analysis. Compared with N1 stage, the presence of N3 was an independent predictor for poor prognosis in OBC cases (HR = 3.955, 95% CI = 2.233–7.006, *p* < 0.001 for BCSS; HR = 2.799, 95% CI = 1.717–4.563, *p* < 0.001 for OS, respectively). HER-2-negative occult breast cancer was shown to be associated with a worse OS (HR = 3.446, 95% CI = 1.211–9.808, *p* = 0.020) but not BCSS. In addition, the lack of radiotherapy was also an independent predictor for worse prognosis in OBC patients (HR = 2.167, 95% CI = 1.306–3.597, *p* = 0.003 for BCSS; HR = 2.164, 95% CI = 1.414–3.311, *p* < 0.001 for OS, respectively).

**Table 6:** Multivariate Cox proportional hazard model of breast cancer-specific survival (BCSS) and overall survival (OS) of OBC patients.

**Variables**		**BCSS**		**OS**	
		**HR (95% CI)**	***P*^**a**^**	**HR (95% CI)**	***P*^**a**^**
Nodal status	1–3	Reference		Reference	
	4–9	1.761 (0.910–3.407)	0.093	1.502 (0.868–2.600)	0.146
	>9	3.955 (2.233–7.006)	**<0.001**	2.799 (1.717–4.563)	**<0.001**
ER status	Positive	Reference		Reference	
	Negative	1.552 (0.816–2.954)	0.181	1.329 (0.780–2.266)	0.295
	Others	4.485 (1.146–17.556)	**0.031**	2.932 (0.803–10.707)	0.104
PR status	Positive	Reference		Reference	
	Negative	2.048 (0.951–4.408)	0.067	1.658 (0.909–3.023)	0.099
	Others	1.067 (0.231–4.919)	0.934	0.895 (0.230–3.481)	0.873
HER-2 status	Positive	Reference		Reference	
	Negative	2.662 (0.916–7.732)	0.072	3.446 (1.211–9.808)	**0.020**
	Others	1.512 (0.529–4.323)	0.440	2.005 (0.713–5.639)	0.187
Radiation	Yes	Reference		Reference	
	No	2.167 (1.306–3.597)	**0.003**	2.164 (1.414–3.311)	**<0.001**

OBC, occult breast cancer; ER, estrogen receptor; PR, progesterone receptor; HER-2, human epidermal growth factor receptor-2; HR, hazard ratio.

a *P-value was adjusted by multivariate Cox proportional hazard regression model including nodal status, ER status, PR status, HER-2 status, radiation. Bold type indicates significance*.

**Figure 4:**
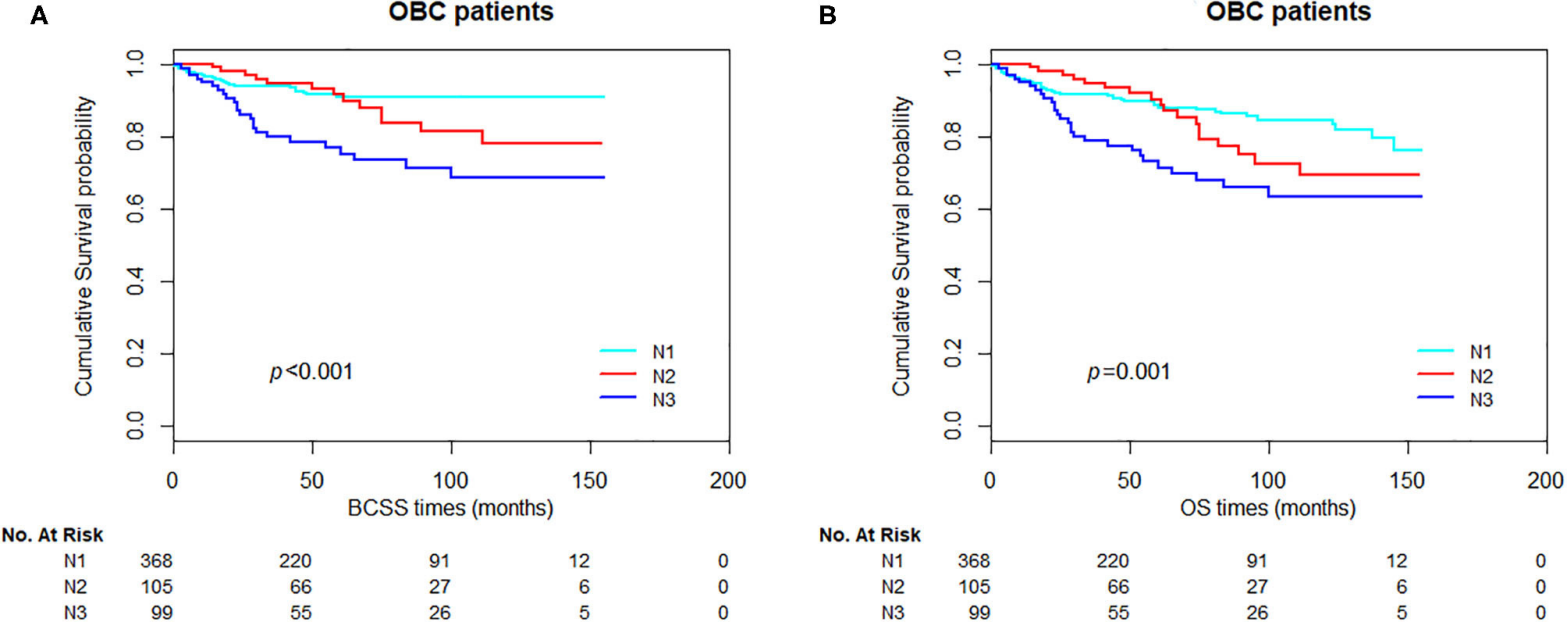
Kaplan–Meier curves of breast cancer-specific survival (BCSS) **(A)** and overall survival (OS) **(B)** based on nodal status for all occult breast cancer (OBC) patients.

**Figure 5:**
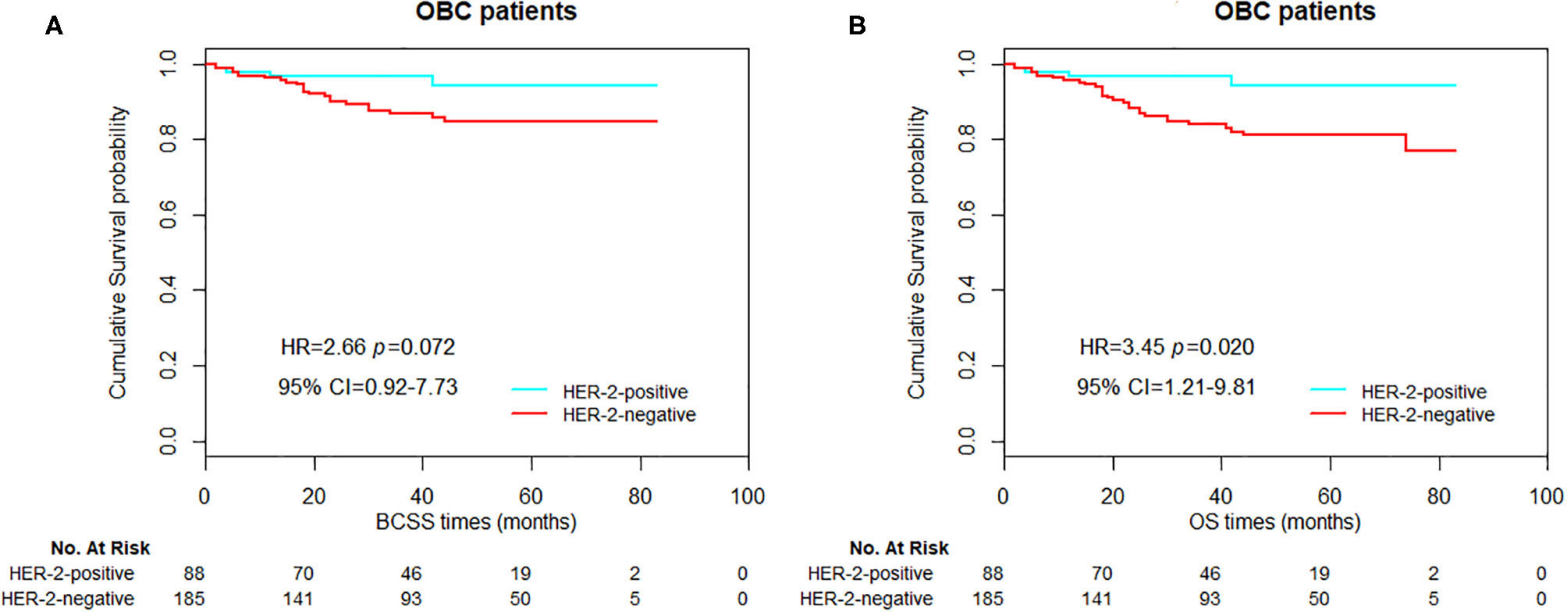
Kaplan–Meier curves of breast cancer-specific survival (BCSS) **(A)** and overall survival (OS) **(B)** based on HER-2 status for all occult breast cancer (OBC) patients.

**Figure 6:**
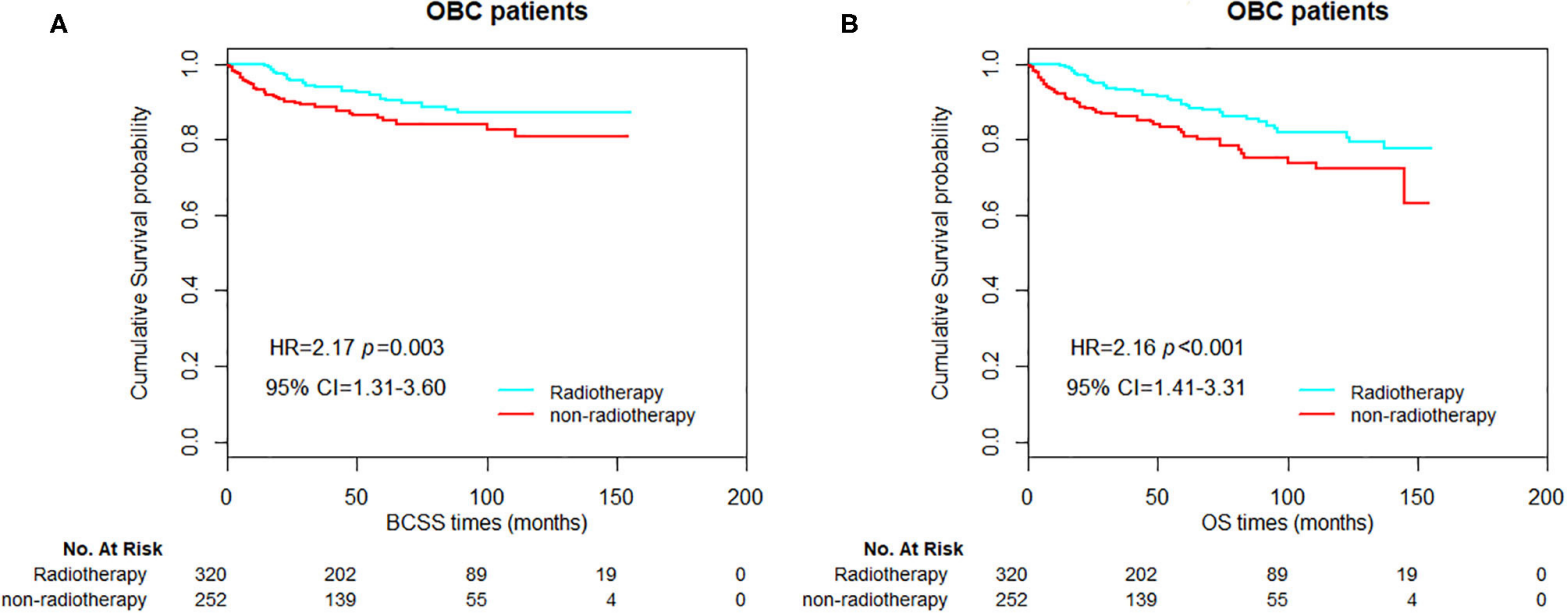
Kaplan–Meier curves of breast cancer-specific survival (BCSS) **(A)** and overall survival (OS) **(B)** based on radiation status for all occult breast cancer (OBC) patients.

In OBC patients, significant differences in BCSS and OS were observed between the groups stratified by nodal status (Figure 4, *p* < 0.001 and *p* = 0.001 for BCSS and OS, respectively). For HER-2-positive and HER-2-negative patients, we could obtain a significant statistical difference in OS, but not BCSS (Figure 5, *p* = 0.072 and 0.020, for BCSS and OS, respectively). As demonstrated in Figure 6, the absence of radiotherapy was associated with worse survival outcome in OBC patients (Figure 6, *p* = 0.04 and 0.008, for BCSS and OS, respectively).

## Discussion

OBC is a rare type of breast cancer. Only few studies have ever evaluated the different clinicopathological characteristics and prognostic factors between OBC and non-OBC patients due to the rarity of these cases. The prognosis of OBC up to now is also controversial. Some studies reported a similar outcome or even worse prognosis for OBC patients compared with non-OBC patients (11, 14, 15). However, in a former study based on SEER database, Ge and colleagues also revealed a survival advantage for OBC patients by using propensity score analysis (13). The other SEER-based study by Johnson et al. (16) assessed the effect of local therapy and other clinical variables on risk of breast cancer-specific mortality in OBC patients. However, these two SEER-based studies mainly focused on the importance of local regional treatment (type of surgery or radiation) in OBC. The clinicopathological characteristics and prognostic factors between OBC and non-OBC patients in different tumor stages and molecular subtypes have not been fully elucidated. Therefore, we conducted this historical cohort study based on SEER database with newest data from 2004 to 2015 and utilize with propensity matching score method, aiming to better demonstrate the different clinicopathological characteristics, and prognostic factors (especially the nodal status and molecular subtypes) between OBC and non-OBC patients.

Totally, 572 cases of OBC and 117,217 cases of non-OBC were selected in this study. Compared with non-OBC patients, the OBC patients appeared to have a higher proportion of older age, N3 stage, ER-negative, PR-negative, and HER2-positive tumors. Additionally, the OBC patients were more likely to receive mastectomy and chemotherapy, with most of these findings were similar to the previous studies (7, 10, 12, 18).

Collectively for all selected cases in our study, multivariate analysis indicated a better survival in overall OBC patients compared with overall non-OBC patients. For patients with N1 or N2 stage, the OBC patients also demonstrated a better prognosis than non-OBC patients. While for patients with N3 stage, no statistical survival differences were observed between OBC and non-OBC patients.

Furthermore, the statistical method of propensity matching score was also conducted in this study to reduce selective bias and minimize the impact of confounding factors. 1144 patients included 572 cases in each subgroup were identified in 1:1 propensity score matched study. No factors differed significantly between OBC and non-OBC patients for matched groups. Compared to overall patients with non-OBC in matched groups, OBC patients revealed a better survival in terms of BCSS and OS, with these survival advantages presented mainly in patients with N2/N3 stage (more than four positive lymph nodes). In general, these survival advantages suggested that OBC had a rather benign biological behavior even if it initially presented as axillary LN metastasis. This could be attributed to a bigger amount of tumor burden in breast lesions of non-OBC patients. It may lead to greater tumor heterogeneity which bring worsen the prognosis of non-OBC patients (19). Additionally, we also compared the matched OBC and non-OBC patients based on different subtypes. For patients with HR-positive tumors, the OBC patients demonstrated a better prognosis than non-OBC patients in terms of BCSS and OS. However, a similar survival was observed between OBC and non-OBC cases in other subtypes. It is well-known that triple-negative breast cancer (TNBC) and HER-2-positive breast cancer possess more mutations than luminal breast cancer (20, 21), thereby leading to a higher risk of recurrence and metastasis in the same treatment conditions and might be the reason why there is no statistical survival difference between OBC and non-OBC patients in other subtypes.

In the cohort of OBC patients, we also sought to investigate the prognostic factors that were related with survival. Univariate Cox regression analysis revealed that AJCC stage, nodal status, ER, PR, or HER-2 status and radiation status significantly correlated with BCSS and OS. However, results from multivariate Cox regression analysis revealed that N3 nodal stage and lack of radiotherapy were two independent prognostic factors for both BCSS and OS. HER-2 negative status only showed an unfavorable prognosis in OS. Nevertheless, we also observed a tendency with poor prognosis in BCSS for patients with ER/PR or HER-2 negative tumor (HR = 1.552, 95% CI = 0.816–2.954, *p* = 0.181, HR = 2.048, 95% CI = 0.951–4.408, *p* = 0.067, and HR = 2.662, 95% CI = 0.916–7.732, *p* = 0.072, respectively). Similar situations were also observed in patients with N2 stage. Compared to patients with N1 stage, patients with 4–9 lymph nodes positive revealed to have a tendency with poor prognosis in BCSS or OS (HR = 1.761, 95% CI = 0.910–3.407, *p* = 0.093, and HR = 1.502, 95% CI = 0.868–2.600, *p* = 0.146, respectively). These may partly due to an limited number of breast cancer-specific events which could not provide sufficient statistical power. Moreover, our analysis suggested that chemotherapy did not improve the outcome of OBC patients. Potential explanations might be a selective bias for retrospective study. Those patients who had a favorable prognosis would rather not to receive chemotherapy while those with high probability of relapse would choose to receive chemotherapy. In addition to this, the exact regimens or cycles of the chemotherapy was also not able to be achieved, thereby resulting in limited data for analysis.

The main advantage of this study is a relatively large sample size of OBC patients based on SEER database and the utilization of propensity matching score method to reduce selective bias and minimize the impact of confounding factors. However, several limitations in this study should also be mentioned. Firstly, our study was based on a retrospective cohort. Although the propensity matching score method was used, a number of potential selection biases might still existed. Secondly, some treatment information such as endocrine therapy or anti-HER2 therapy is not available in SEER, while these information were critically essential for the survival outcome for node-positive breast cancer. Thirdly, a lack of central pathology review should be also acknowledged in our study. Additional population-based and multi-institutional studies with larger sample size are warranted.

Up to now, the treatment of OBC almost completely referred to non-OBC. Our study demonstrated that OBC patients had a better outcome compared to non-OBC patients, especially for those with N2/N3 stage or HR-positive tumors. Therefore, de-escalation therapy might be appropriate for selected OBC patients. Nodal status, HER-2 status, and radiation status were three independent prognostic factors for OBC patients. These results not only provided further understanding of OBC but also contributed to clinical practice that clinicians might provide improve clinical management for OBC patients.

## Data Availability Statement

The raw data supporting the conclusions of this article will be made available by the authors, without undue reservation.

## Author Contributions

C-GS contributed to conception and design. K-YH, JZ, W-FF, and Y-XL contributed to the development of methodology. K-YH and JZ contributed to acquisition of data and analysis and interpretation of data. K-YH, JZ, Y-XL, and C-GS wrote, reviewed, and/or revised the manuscript. C-GS did study supervision. All authors contributed to the article and approved the submitted version.

## Conflict of Interest

The authors declare that the research was conducted in the absence of any commercial or financial relationships that could be construed as a potential conflict of interest.
