# Supplementary material for: Different Clinicopathological Characteristics and Prognostic Factors for Occult and Non-occult Breast Cancer: Analysis of the SEER Database
Source: Front Oncol. 2020 Aug 19;10:1420. doi: 10.3389/fonc.2020.01420 (PMC7466661; doi:10.3389/fonc.2020.01420)
Supplement: Supplementary file 1 [file Table_1.DOC]

**Supplementary Table S1** Univariate Cox proportional hazard model of breast cancer-specific survival (BCSS) and overall survival (OS) of OBC patients

| **Variables** | | **BCSS** | |  | **OS** | |
| --- | --- | --- | --- | --- | --- | --- |
|  |  | **HR (95 % CI)** | **P** |  | **HR (95 % CI)** | **P** |
| **Year of diagnosis** | **2004-2009** | 0.842 (0.504–1.406) | 0.511 |  | 0.852 (0.539–1.345) | 0.491 |
|  | **2010-2015** | Reference |  |  | Reference |  |
| **Age (years)** | **20–49** | 0.910 (0.511–1.618) | 0.747 |  | 0.731 (0.437–1.223) | 0.233 |
|  | **50–79** | Reference |  |  | Reference |  |
| **Race** | **White** | Reference |  |  | Reference |  |
|  | **Black** | 0.861 (0.410–1.807) | 0.692 |  | 1.032 (0.572–1.860) | 0.918 |
|  | **Other ^a^** | 0.589 (0.184–1.884) | 0.373 |  | 0.858 (0.373–1.973) | 0.719 |
| **Marital status** | **Married** | Reference |  |  | Reference |  |
|  | **Not married ^b^** | 1.396 (0.861–2.262) | 0.176 |  | 1.435 (0.954–2.156) | 0.083 |
| **Grade** | **I and II** | Reference |  |  | Reference |  |
|  | **III and IV** | 1.616 (0.484–5.402) | 0.435 |  | 1.539 (0.541–4.380) | 0.419 |
|  | **Unknown** | 0.977 (0.302–3.155) | 0.969 |  | 1.058 (0.384–2.911) | 0.914 |
| **Laterality** | **Left** | Reference |  |  | Reference |  |
|  | **Right** | 0.722 (0.440–1.183) | 0.196 |  | 0.789 (0.522–1.191) | 0.260 |
| **Stage** | **II** | Reference |  |  | Reference |  |
|  | **III** | 2.322(1.428-3.776) | **0.001** |  | 1.793(1.194-2.693) | **0.005** |
| **Nodal status** | **1 to 3** | Reference |  |  | Reference |  |
|  | **4 to 9** | 1.520 (0.790–2.924) | 0.210 |  | 1.315 (0.764–2.265) | 0.323 |
|  | **> 9** | 3.251 (1.893–5.585) | **<0.001** |  | 2.340 (1.465–3.736) | **<0.001** |
| **ER status** | **Positive** | Reference |  |  | Reference |  |
|  | **Negative** | 2.156 (1.261–3.686) | **0.005** |  | 1.666 (1.078–2.574) | **0.022** |
|  | **Others** | 2.800 (1.326–5.913) | **0.007** |  | 1.819 (0.928–3.567) | 0.082 |
| **PR status** | **Positive** | Reference |  |  | Reference |  |
|  | **Negative** | 2.522 (1.334–4.768) | **0.004** |  | 1.818 (1.115–2.964) | **0.016** |
|  | **Others** | 2.456 (1.034–5.830) | **0.042** |  | 1.597 (0.774–3.296) | 0.205 |
| **HER-2 status** | **Positive** | Reference |  |  | Reference |  |
|  | **Negative** | 2.704 (0.935–7.821) | 0.066 |  | 3.505 (1.235–9.949) | **0.018** |
|  | **Others** | 1.808 (0.639–5.120) | 0.265 |  | 2.182 (0.780–6.104) | 0.137 |
| **Breast subtype** | **HR-/Her2+** | 1.609 (0.504–5.131) | 0.442 |  | 1.009 (0.337–3.018) | 0.988 |
|  | **HR+/Her2+** | N/A |  |  | N/A |  |
|  | **HR+/Her2-** | Reference |  |  | Reference |  |
|  | **Triple negative** | 2.168 (0.950–4.945) | 0.066 |  | 1.445 (0.705–2.962) | 0.314 |
|  | **Unknown** | 0.955 (0.469–1.945) | 0.900 |  | 0.723 (0.405–1.292) | 0.273 |
| **Mastectomy** | **Yes** | Reference |  |  | Reference |  |
|  | **No** | 1.335(0.790–2.257) | 0.281 |  | 1.402(0.895–2.194) | 0.140 |
| **Chemotherapy** | **Yes** | Reference |  |  | Reference |  |
|  | **No/unknown** | 0.874(0.446-1.713) | 0.695 |  | 1.149(0.687-1.923) | 0.597 |
| **Radiation** | **Yes** | Reference |  |  | Reference |  |
|  | **No** | 1.724 (1.062–2.799) | **0.028** |  | 1.799 (1.194–2.708) | **0.005** |

Abbreviation: OBC, occult breast cancer; ER, estrogen receptor; PR, progesterone receptor; HR, hormone receptor; HER-2, human epidermal growth factor receptor 2; HR, hazard ratios; CI, conﬁdence interval; N/A, Not available; BCSS, breast cancer-specific survival; OS, overall survival.

^a^ Other includes American Indian/Alaskan native and Asian/Paciﬁc Islander.

^b^ Not married includes divorced, separated, single (never married), unmarried or domestic partner and widowed.
